# Simulation-Based Identification of Operating Point Range for a Novel Laser-Sintering Machine for Additive Manufacturing of Continuous Carbon-Fibre-Reinforced Polymer Parts

**DOI:** 10.3390/polym15193975

**Published:** 2023-10-03

**Authors:** Michael Baranowski, Zijin Shao, Alexander Spintzyk, Florian Kößler, Jürgen Fleischer

**Affiliations:** 1 Institute of Production Science, Faculty of Mechanical Engineering, Karlsruhe Institute of Technology (KIT), Kaiserstaße 12, 76131 Karlsruhe, Germany; taopiangearen@gmail.com (Z.S.);; 2 Karlsruhe Research Factory, Karlsruhe Institute of Technology (KIT), Rintheimer Querallee 2, 76131 Karlsruhe, Germany

**Keywords:** laser sintering (LS), continuous carbon-fibre-reinforced polymer parts (CCFRP), fibre integration unit, heat-affected zone, finite element model, central composite design (CCD)

## Abstract

Additive manufacturing using continuous carbon-fibre-reinforced polymer (CCFRP) presents an opportunity to create high-strength parts suitable for aerospace, engineering, and other industries. Continuous fibres reinforce the load-bearing path, enhancing the mechanical properties of these parts. However, the existing additive manufacturing processes for CCFRP parts have numerous disadvantages. Resin- and extrusion-based processes require time-consuming and costly post-processing to remove the support structures, severely restricting the design flexibility. Additionally, the production of small batches demands considerable effort. In contrast, laser sintering has emerged as a promising alternative in industry. It enables the creation of robust parts without needing support structures, offering efficiency and cost-effectiveness in producing single units or small batches. Utilising an innovative laser-sintering machine equipped with automated continuous fibre integration, this study aims to merge the benefits of laser-sintering technology with the advantages of continuous fibres. The paper provides an outline, using a finite element model in COMSOL Multiphysics, for simulating and identifying an optimised operating point range for the automated integration of continuous fibres. The results demonstrate a remarkable reduction in processing time of 233% for the fibre integration and a reduction of 56% for the width and 44% for the depth of the heat-affected zone compared to the initial setup.

## 1. Introduction

The utilisation of continuous carbon-fibre-reinforced polymer (CCFRP) parts in industrial applications presents a substantial opportunity for achieving significant reductions in future product consumption and CO2 emissions while maintaining economic viability [1]. CCFRP parts are notable for their favourable weight-to-strength ratio and impressive mechanical tensile properties along the fibre orientation. Continuous fibres play a crucial role in enhancing the mechanical characteristics of fibre-reinforced parts along the load-bearing pathway [2].

Additive manufacturing processes offer a promising avenue for the tool-less and time-efficient production of CCFRP parts, allowing for high levels of customisation and shape complexity. Material extrusion (MEX), which includes techniques like fused layer modelling (FLM) and ARBURG plastic freeforming (APF), has gained prominence in the literature as a viable method for the additive manufacturing of CCFRP parts [3,4,5,6,7,8,9]. Another category of processes employed for CCFRP parts is vat photopolymerisation (VPP) [10,11,12]. However, it is essential to note that CCFRP parts produced using these processes (MEX and VPP) have decisive disadvantages. The inherent nature of these processes necessitates using support structures, which must be removed and managed after production. This results in additional time and cost implications for the disposal and post-processing steps. Furthermore, the reliance on support structures restricts the ability to create features such as overhangs, cavities, and undercuts, consequently limiting the complexity of the produced parts. Additionally, removing support structures can potentially introduce surface defects to the remaining part surfaces, leading to a less uniform appearance of the parts. Moreover, MEX and VPP processes do not facilitate cost-effective small-batch production.

In contrast, the laser-sintering (LS) process is a compelling alternative for CCFRP parts. In a comparative analysis of the mechanical and thermal properties, as well as the long-term stability of polymer parts produced via material extrusion (MEX), vat photo polymerisation (VPP), and LS, the LS process exhibits notable advantages, notably in the creation of durable, functional parts akin to those produced through injection moulding [13,14]. As reported in [13], laser-sintered specimens demonstrate a higher Young’s modulus compared to their injection-moulded counterparts, attributed to the increased crystallinity in the molecular structure of the semi-crystalline thermoplastics. The tensile strength in the LS-produced specimens closely approximates that of their injection-moulded counterparts. Additional merits of the LS process include the absence of support structures, offering greater design flexibility. In LS, unprocessed powder is a natural support structure, eliminating the need for labour-intensive and costly post-processing steps [15]. Furthermore, LS can generate near-net-shape functional parts with intricate features like undercuts, cavities, and overhangs in a single manufacturing step. The LS process’s ability to efficiently position parts vertically and horizontally within the powder bed facilitates cost-effective small-batch production [13,16]. Compared to parts produced through fused layer modelling (FLM), LS-produced parts exhibit significantly reduced anisotropy, enhanced dimensional accuracy, and reduced surface roughness [13,17]. Consequently, the LS process offers promising foundational properties (matrix) for CCFRP parts. However, it is worth noting that there are currently no commercially available LS machines that fully integrate the advantages of the LS process with those of continuous fibres. Challenges associated with integrating continuous fibres into the LS process primarily revolve around intricate temperature control and the repetitive recoater movement for each layer.

To harness the distinct advantages of the LS process and leverage the promising attributes of continuous carbon fibres, a technical demonstration of feasibility was conducted [18,19,20]. This involved the layer-wise integration of continuous rovings (fibre strands) into laser-sintered parts composed of PA12, facilitated by a newly developed LS machine. Central to this prototype LS machine is a dedicated fibre integration unit designed to seamlessly incorporate the rovings into the pre-existing layers of the parts. A heated fibre nozzle is employed to liquefy the polymer locally, creating a heat-affected zone (HAZ) characterised by specific width and depth parameters. Subsequently, a synchronised series of movements involving the roving feed rate and the nozzle feed rate is used to introduce the roving into the liquefied polymer melt. In a prior study [19], an in-depth examination of the extended LS process was conducted, focusing on temperature control during the integration of fibres to determine the optimal operational conditions for achieving curl-free roving integration. Furthermore, an investigation into the impact of the process parameters and their interactions on the dimensions of the HAZ was carried out [18] using a split-plot design (SPD). The findings from these previous studies [18,19] served as the foundational knowledge for this research. However, the insights gained from [18] led to an inefficient production process for continuous carbon-fibre-reinforced polymer CCFRP parts. The production speed was relatively slow, necessitating a low feed rate for roving integration. This low feed rate resulted in an economically disadvantageous and time-consuming production of CCFRP parts. Additionally, the dimensions of the HAZ, particularly its width and depth, were more significant than desired, requiring the placement of rovings at a significant distance from the part edges. This oversized HAZ resulted in a limitation in achieving a higher fibre volume content (FVC) and the corresponding mechanical properties. As a result, the full potential of the new LS process with continuous fibre integration remained untapped. Only by identifying an optimised operational point for roving integration is it possible to reduce the dimensions of the HAZ and, crucially, the processing time in a targeted manner. A systematic approach to determining an operating point that ensures a secure process and a suitably compact HAZ while enabling swift roving integration is imperative for the cost-effective production of LS components with elevated FVC and enhanced mechanical properties.

Therefore, the objective of this paper is the simulation-based identification of an optimal operating point range within which the smallest possible HAZs can be generated, and the rovings can be integrated into the part as quickly as possible but simultaneously in a process-safe manner. Using COMSOL Multiphysics (Version 6.1), the process zone is first modelled with the help of a macroscopic modelling approach. Using this FE model, the creation process of the HAZ, due to the heat input of the heated fibre nozzle, is to be simulated, and an optimised operating point range is to be identified in a subsequent simulation study. Section 2.1 presents the principle of roving integration. The influencing and target variables on the roving integration caused by the fibre integration unit are discussed in the same section. The procedure for deriving the FE model in COMSOL (Version 6.1) is described in Section 2.2. The same section presents the evaluation procedure for evaluating the FE model’s accuracy using a convergence analysis and a plausibility check. Section 2.3 describes the approach for the simulation-based identification of an optimised operating point range with the help of a central composite design (CCD) with initially assumed factor levels within which an optimised operating point range is sought. Finally, the identified operating point range derived by the FE model is experimentally validated, and the roving integration is analysed in the same chapter using an adjusted, more detailed CCD. Section 3 presents and discusses the results concerning the initial state.

This study successfully showcased a substantial 233% reduction in the processing time required for roving integration. Consequently, this achievement paves the way for the more cost-effective production of CCFRP parts using the developed LS machine. Furthermore, 56% and 44% reductions in the width and depth of the heat-affected zone (HAZ) were attained. This advancement enables the integration of rovings closer to the edges of the parts, thereby permitting a higher fibre volume content (FVC). As a result, this study offers optimised operation points that can guide future research efforts in systematically enhancing the FVC and the accompanying mechanical properties of CCFRP parts.

## 2. Materials and Methods

### 2.1. Principle of Roving Integration

**Figure 1:**
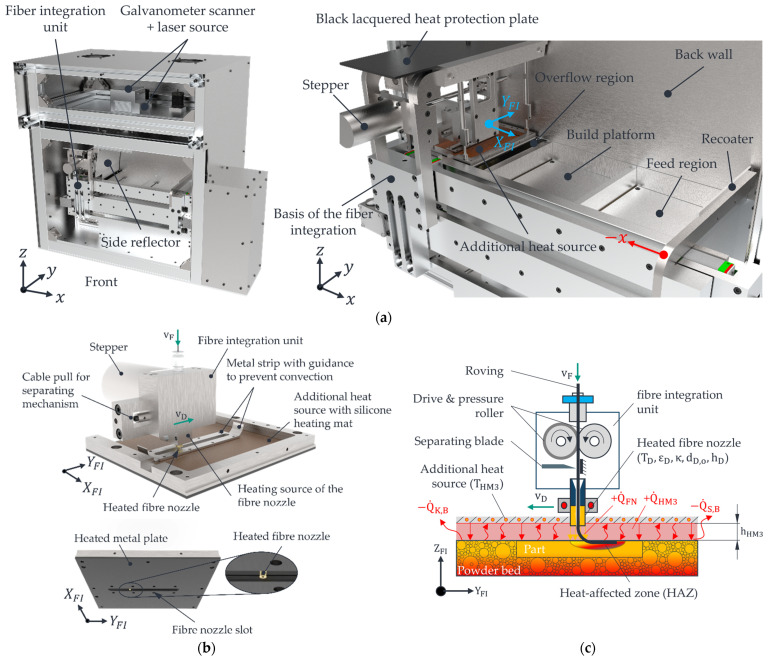
View of the process chamber of the developed LS machine (**a**) with a detailed view of the fibre integration unit (**b**). Schematic representation of the heat fluxes and influencing factors (**c**).

This section outlines the process flow to provide a fundamental understanding of roving integration within the developed LS machine—see Figure 1a.

It is important to note that this paper does not offer an exhaustive description of the machine itself or the achievable properties of the produced parts, as those details can be found in [18,20]. The process begins within a heated process chamber of the LS machine, maintained at approximately 110 °C. Subsequently, a fresh layer of powder is evenly applied by the recoater, and the powder bed’s surface temperature is homogenised using infrared (IR) emitters. Black PA12 powder (Sintratec AG, Brugg, Switzerland) with a melting temperature of approx. 185 °C is used as the matrix material. The laser beam then liquefies the applied powder layer. Following ISO 6983 G-code instructions, the layer-specific (2D) integration of one or more rovings is carried out sequentially. In order to be able to process parts with the developed LS machine, a MATLAB app was developed in xy to slice the parts (.stl) and generate the G-code for roving integration [21]. The developed LS machine uses 1K rovings (HTA40) with 67 tex (Teijin Limited, Tokyo, Japan), a width of about 365 µm and a thickness of about 110 µm. The 1K rovings have an elliptical shape in their delivery condition. To facilitate this process, the entire fibre integration unit, with an additional heat source and a heated fibre nozzle, is moved rapidly in the x and y directions to reach the initial point of the first roving path located in the stable sintering region. Detailed descriptions of all symbols used in this process can be found in Table 1 and Table 2.

Detailed illustrations of the fibre integration unit and its constituent components are provided in Figure 1b,c. Throughout the entire roving integration process, the structure of the fibre integration unit moves into the path of radiation from the infrared (IR) emitters installed within the LS machine. This movement results in the shadowing of IR radiation. To maintain the powder bed’s part and surface within the sintering window, a metal plate fitted with an adhered silicone heating mat (functioning as an additional heat source) is positioned parallel to the powder surface. It is situated at a distance of $h_{\mathrm{HM}3\;}\;$beneath the bottom side of the fibre integration unit. The term “sintering window” refers to a temperature range encompassing the interval between the onset of crystallisation and the melting of the semi-crystalline thermoplastic material used. The PA12 material employed in this study corresponds to an approximate crystallisation temperature of 154 °C and a melting temperature of around 184 °C, resulting in a sintering window of 30 °C [19,22]. The interaction between the heated fibre nozzle, operating at temperature$\;T_{\mathrm{HM}3\;}$, and the part’s surface induces the formation of a localised melt zone or heat-affected zone (HAZ). The dimensions of this HAZ, namely, its width $b_{\mathrm{HAZ}}$ and depth${\;t}_{\mathrm{HAZ}}$, describe the extent to which the polymer’s viscosity is locally reduced. The successful embedding of rovings within the part relies on creating a sufficiently deep HAZ through the action of the heated fibre nozzle. The resultant molten material adheres to the roving, firmly anchoring it to the underlying layers. Positioned above the fibre nozzle, a cutting blade trims the continuous roving to a length specified by the programmed instructions in the G-code. It is important to note that the built-in diode laser, operating at 450 nm with a power output of 1.6 W, remains inactive during the roving integration process. Following the successful integration of the rovings, the fibre integration unit returns to its designated home position, and the recoater applies a fresh layer of powder. Once the infrared (IR) emitters have sufficiently heated and homogenised the powder bed surface to reach the sintering temperature of approximately 175 °C, the laser is employed to melt the new powder layer, thereby fully incorporating the roving within the polymer matrix. This sequence is repeated until all rovings have been integrated per the instructions specified in the G-code. Subsequently, after the printing process concludes, a controlled cooling process is initiated for the powder bed housing the CCFRP parts.

#### 2.1.1. Transferred Heat with Influencing and Target Variables during Roving Integration

An analytical examination of the heat transfer process is employed to determine the variables that have an affect and those are aimed for in the context of roving integration. One-dimensional heat flows were utilised to simplify this analysis to characterise the influencing and target variables outlined in this study. The heat transfer during the roving integration process can be mathematically described in Equation (1).

(1) $$Q_{\mathrm{FI}}\left[J\right]={\;Q}_{\mathrm{HM}3}+Q_{\mathrm{FN}}$$

The first term in Equation (1) accounts for the heat delivered to the surface of the powder bed via the additional heat source. The part and the powder bed surface are kept warm within the sintering window, with the assistance of the additional heat source denoted as $Q_{\mathrm{HM}3}$, located on the bottom side of the fibre integration unit to ensure the integration of rovings in a secure and replicable manner. For an extensive analysis of the additional heat source and $Q_{\mathrm{HM}3}$, please refer to [19].

The second term in Equation (1) represents the heat the heated fibre nozzle conveys. This heat flow primarily plays a pivotal role in directing heat energy transfer, thus facilitating the formation of the HAZ. The heat transferred through the fibre nozzle is quantified according to Equation (2).

(2) $$Q_{\mathrm{FN}}=\;\pi \cdot \left(r_{D,o}^{2}-r_{D,i}^{2}\right)\cdot \left(\lambda_{L}\cdot \frac{T_{D}-T_{O}}{h_{D}}+\frac{\sigma }{\frac{1}{\varepsilon_{D}}+\frac{1}{\varepsilon_{P}}-1}\;\cdot \left(T_{D}^{4}-T_{O}^{4}\right)\right)\cdot \Delta t_{\mathrm{FN}}$$

The first term within the parentheses in Equation (2) pertains to Fourier’s law, describing the heat exchange between the ring-shaped fibre nozzle characterised by inner diameter $d_{D,i\;}$(responsible for guiding and transmitting the roving inside the fibre nozzle), outer diameter $d_{D,o},\;$nozzle curvature κ, and the surface of the powder bed. It is assumed that heat is exclusively transferred through heat conduction across the air gap$\;h_{\mathrm{HM}3}$, which constitutes a scenario of free convection involving internal flow and heat radiation [18,20]. Under these conditions, the Rayleigh number (Ra) is less than 1, indicating a stable stratified fluid layer with no induced flow (Nusselt number, Nu, equals 1) [23]. The second term within the parentheses in Equation (2) corresponds to the Stefan–Boltzmann law, incorporating an additional radiation exchange between the front surface of the fibre nozzle and the surface of the powder bed. From the standpoint of a stationary point on the part’s surface, $\Delta t_{\mathrm{FN}}$ signifies the temporal duration during which the heated fibre nozzle with a surface area $\;A_{D}\left(d_{D,i},d_{D,o}\right)$ imparts heat to the part at a feed rate of ${\;v}_{D\;}$. The duration $\Delta t_{\mathrm{FN}}\;$ for heat transfer can be influenced by the feed rate $v_{D}$ of the fibre nozzle. It is important to note that potential convection currents between the fibre nozzle and the supplementary heat source and heat dissipation at the peripheries of the additional heat source into the surroundings were assumed but not considered in this analysis.

The parameters outlined in Equation (2) summarise the factors influencing the roving integration process within the developed laser-sintering machine, and their definitions are provided in Table 1. It is imperative to mention that this analysis did not incorporate influences arising from the laser-sintering process, such as interactions between the laser and the part, material composition, or ageing effects of the powder. These factors were maintained as constant as possible throughout the studies, as detailed in Section 2.3.

**Figure 2:**
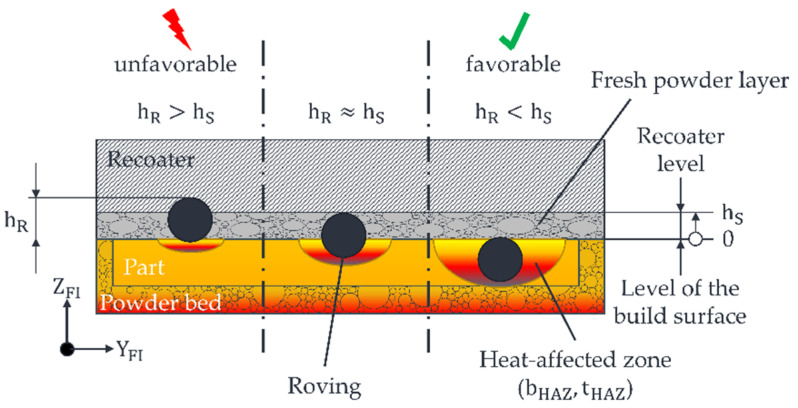
Schematic representation of the influence of the HAZ on the roving overlap ${\;h}_{R}$.

The target variables for successfully integrating rovings in the developed LS machine are depicted in Figure 2.

It is crucial to create a HAZ to position the roving below the recoater’s movement level, denoted as $h_{S}\;$ (the predetermined layer thickness during the printing process), to ensure the successful integration of rovings. In practical terms, $t_{\mathrm{HAZ}\;}\;$ needs to be adjusted to sufficiently immerse the roving in the molten material, meaning $h_{R\;}\;$should be less than ${\;h}_{S}$. This configuration avoids the formation of any disruptive contours for the recoater, ensuring process reliability.

Consequently, the finite element (FE) model must yield a value for ${\;t}_{\mathrm{HAZ}}\;$ corresponding to a roving thickness of approximately 365 µm. If the roving extends too far beyond the part’s edges $\;h_{R}\geq h_{S}$, it could lead to a collision between the recoater and the roving during the subsequent recoating process. Such an occurrence may result in the part being displaced by the recoater, necessitating the stop of the printing process. Additionally, minimising ${\;b}_{\mathrm{HAZ}}\;$ is essential for enabling rovings to be placed as close to the edges of the part as possible without causing the unsintered and loose powder to melt beyond its edges. According to the results of the SPD from [21], $b_{\mathrm{HAZ}}$ and ${\;t}_{\mathrm{HAZ}}$ of the HAZ constantly change with a constant aspect ratio ${\;b}_{\mathrm{HAZ}}\;$/${\;t}_{\mathrm{HAZ}}\;$ when varying the process parameters (e.g., fibre nozzle feed rate and nozzle temperature). In other words, if, for example, ${\;t}_{\mathrm{HAZ}}\;$of the HAZ is reduced by increasing the nozzle feed rate, ${\;b}_{\mathrm{HAZ}}\;$is reduced simultaneously. For this reason, only ${\;t}_{\mathrm{HAZ}}$ is considered in this paper, as this is primarily responsible for the process reliability of the roving integration. Regarding cost-effective production, the duration required for roving integration plays a crucial role. This processing time is determined by the feed rate $v_{D}$ of the fibre nozzle. The specific objectives for successful fibre integration are outlined in Table 2.

Initial studies were conducted in [18,19] for the influencing and target variables listed in Table 1 and Table 2. The experimental analysis and quantification of the effects and interactions of ${\;v}_{D}$,${\;h}_{D}$,${\;d}_{D,o}$,${\;h}_{\mathrm{HM}3}$, κ, and $T_{D}$ on the width and depth of the HAZ (without the influence of roving integration) were analysed using an SPD in [18,24]. According to [18], $v_{D}$,${\;h}_{D}$,${\;d}_{D,o}\;$, and $T_{D}$ have the most significant influence on the shape of the HAZ. $h_{D}$ cannot be set smaller than 0.6 mm from the powder bed surface; otherwise, the powder can adhere to the fibre nozzle and, thus, reduce the effective fibre nozzle distance. This leads to entrainment effects in the powder bed or the part. Furthermore, an outer diameter of the fibre nozzle of 2 mm has been established. $d_{D,o}$ directly influences the ${\;b}_{\mathrm{WEZ}}$. Due to ${\;d}_{D,i}=$ 0.6 mm, the outer diameter cannot be reduced further, as this would weaken the fibre nozzle too much. According to [19], an operating point was found for $T_{\mathrm{HM}3}$ and $h_{\mathrm{HM}3}$ at $T_{\mathrm{HM}3}=$ 190 °C and $h_{\mathrm{HM}3}=$ 0.8 mm, where rovings can be reliably integrated into the parts. This leaves $T_{D}$ and $v_{D}$ for an optimisation of $t_{\mathrm{WEZ}}$. In other words, the two factors, $T_{D}$ and $v_{D}$, can be varied to optimise $t_{\mathrm{WEZ}}$. According to the SPD in [19] and Equation (2), a further reduction of $t_{\mathrm{WEZ}}$ is achieved by increasing $T_{D}$ and $v_{D}$ as optimisation direction.

This paper aims to determine an operating point range in which the condition $t_{\mathrm{HAZ}}\;\geq$ 365 µm is achieved with the highest possible fibre nozzle feed rate $v_{D}\;$using an FE model in COMSOL Multiphysics (Version 6.1). The findings from the SPD in [19] are used to evaluate the FE model’s plausibility and accuracy with the help of a CCD with initially assumed factor levels within which an optimised operating point range ($t_{\mathrm{HAZ}}\;\geq$ 365 µm) is sought. Finally, the adjusted operating point range derived by the FE model is validated with the help of an adapted and experimentally performed CCD with more detailed factor levels. In addition, the influence of roving integration on the depth of the HAZ is analysed, and an optimised operating point for roving integration is experimentally derived. The results are described and discussed in Section 3.

### 2.2. Numerical Modelling

In this section, a systematic derivation of an FE model for modelling the formation process of the HAZ is carried out based on physical assumptions and simplifications. The results generated from the FE model are first compared with the results of the SPD from [18] to evaluate the plausibility and accuracy of the FE model. The FE model was then used to identify an optimal operating point range for $T_{D}$ and ${\;v}_{D}$.

#### 2.2.1. Modelling Approach

A macroscopic modelling approach is employed in which individual particles within the part are not individually considered. Instead, the molten part is treated as a continuous medium with homogenised properties. The fluctuations in temperature within the molten part due to the heat source $Q_{\mathrm{FI}}\;$ are described using a nonlinear heat transfer equation as defined in Equation (3) [25].

(3) $$\rho_{P}c_{P}\frac{\partial T}{\partial t}=-\nabla q+Q$$

The quantities $\rho_{P}$ $\left[\mathrm{kg}/m^{3}\right]\;$and $C_{P}\;\left[J/\left(\mathrm{kg}\cdot K\right)\right]$ represent the part’s density and heat capacity. Equation (3) delineates the alterations in the part’s temperature resulting from the heat fluxes q $\left[W/m^{2}\right]$ and external heat sources  Q $\left[W/m^{3}\right]$, predominantly involving conduction, convection, and radiation.

#### 2.2.2. Model Assumptions and Simplifications

For the derivation of the FE model, some physical assumptions and simplifications are made based on the findings from [18,19] to keep the computing time and the required storage space low while still gaining maximum knowledge. The most important assumptions and simplifications are listed in the following points.

- The supplied heat flow $Q_{\mathrm{HM}3}$ compensates for heat losses due to radiation and convection according to Equation (1), consisting of heat conduction through the air gap and the radiation exchange between the installed additional heat source and the part surface. Consequently, the heat losses are not considered in the FE model but only the supplied heat $Q_{\mathrm{HM}3}$ of the additional heat source.
- According to Equation (2), the heat input is based on heat conduction through the air gap and the radiation exchange between the ring-shaped fibre nozzle and the part’s surface. The FE model does not consider possible convection flows between the fibre nozzle and the additional heat source.
- The surrounding and loose powder bed is not considered in the FE model, but only the already manufactured part. The part edges are isolated in the FE model.
- Modelling approaches from the current state of research and technology for analysing the laser-sintering process consider the phase transformation from powdery to molten states [26,27]. However, in the developed LS machine, the HAZ is generated in the molten state of the part, which the laser has transformed. The phase transition has, therefore, already taken place when the HAZ was created. Therefore, only the material properties of the molten state of the part are used. Isotropic part properties are assumed. This means that constant values are used for the thermal conductivity of the part ${\;\lambda }_{P}$ for the part density $\rho_{P}$, and, thus, for the porosity of the part $\Phi_{P}$ and the specific heat capacity $c_{P}$ (constant pressure). The thermal conductivity of the air in the air gap is the only variable with a temperature dependence ${\;\lambda }_{A}\left(T\right)$.
- To determine the target value $t_{\mathrm{HAZ}}$ in the FE model, only the part area with a temperature value equal to or higher than the melting temperature of the PA12 part $T_{M}$ is evaluated.
- According to Figure 1b, there is symmetry in the centre of the fibre nozzle and along the y-axis (axis of movement of the fibre nozzle). Due to this symmetry property, only half of the process zone is modelled in the FE model.
- To simplify the FE model, not the entire additional heat source is modelled, but only the immediate vicinity of the process zone, consisting of the PA12 part, fibre nozzle, air gap, feeler gauge tape, metal plate of the additional heat source, and guidance.
- Due to the roving’s current scattering position/orientation in part [18], only the formation process of the HAZ is modelled. The influence of the roving on the HAZ is, therefore, not an object of investigation of the FE model. For a simulation-based identification of an operating point range with the highest possible fibre nozzle feed rate, a value of $t_{\mathrm{HAZ}}\approx$ 365 µm is assumed. According to Table 1, this corresponds to the approximate thickness of a 1K roving.

#### 2.2.3. Geometric Model Structure

**Figure 3:**
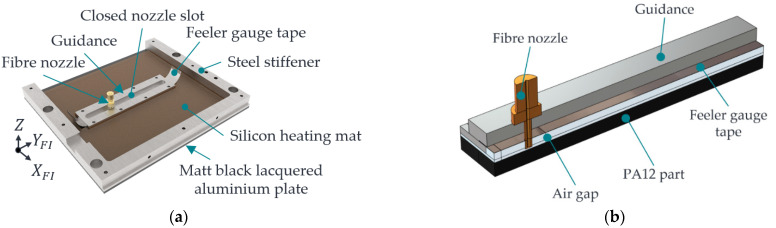
Three-dimensional view of the additional heat source with fibre nozzle, feeler gauge tape, and guide (**a**), and geometry implemented in COMSOL for process zone (**b**).

To build an FE model in COMSOL Multiphysics (Version 6.1), a geometric representation of the process zone to be investigated must first be created. According to Section 2.1.1, the process zone consists of the PA12 part in which the HAZ is generated, along with the air gap, the feeler gauge tape with guidance, and the fibre nozzle, which is mandatory for creating the HAZ. These components are modelled within COMSOL so that the settings to be investigated can be parameterised. A parameterisation of the geometry parameters ($h_{D}$,${\;h}_{\mathrm{HM}3}$,${\;d}_{D,o}$, and κ) enables a simple and automated variation of the influencing variables using a MATLAB script linked to the FE model when carrying out parameter studies. Figure 3a shows the CAD model, including the installed components of the process zone. For comparison, Figure 3b shows the simplified geometry of the process zone realised in COMSOL.

#### 2.2.4. Material Properties with Initial Settings

Table 3 shows the material parameters relevant to the FE model with numerical values and sources. In addition, Table 3 shows the initial values for all materials that form the starting point for the simulation.

As described in Section 2.2.2, isotropic material properties are assumed for the PA12 part. For modelling heat conduction through the air gap, the heat conduction coefficient through the air is relevant. The temperature-dependent thermal conductivity can be approximated according to [23] using Equation (4).

(4) $$\lambda \left(T\right)=0.0651T+24.881$$

#### 2.2.5. Meshing Zones and Moving Mesh

**Figure 4:**
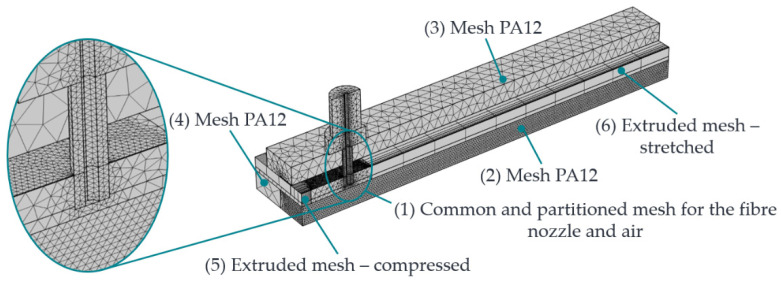
Process zone with the occurring mesh zones and initially assumed mesh resolution.

To reduce the calculation time, the individual meshing zones of the assembly are meshed with different degrees of resolution according to their expected influence on the simulation results. For this reason, the geometry from Figure 3 is partitioned into sections. The following Figure 4 shows the meshing zones used.

Table 4 lists the individual meshing zones with the initially assumed mesh resolution. The meshing parameters for the individual mesh resolutions are in the COMSOL documentation [33].

**Figure 5:**
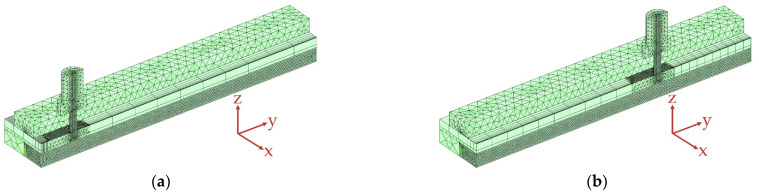
The initial state of the moving mesh at time t = 0 s (**a**) and the extruded mesh at a relative fibre nozzle offset of 40 mm (**b**).

For a detailed description of the mesh specifications, please refer to [33]. The PA12, the front surface of the nozzle, and the air near the nozzle are significantly involved in the heat transfer and the formation of the HAZ. Consequently, a fine mesh is initially assumed there. The nozzle and the air section mesh in a standard mesh using a parameterisable selection cylinder along the rotation axis of the fibre nozzle in COMSOL. The PA12 part is also partitioned. In this way, a correspondingly fine mesh can be selected in the area where the HAZ is formed, and the low-influence areas of the PA12 part can be more coarsely meshed. The guidance, the feeler gauge tape, and the black lacquered metal plate are provided with a coarser mesh. By default, geometries in COMSOL are meshed tetrahedrally. To replicate the one-dimensional movement of fibre nozzle feed along the y-direction, the moving mesh knot is used in COMSOL. The moving mesh knot allows the deformation of the extruded meshes (zones 5 and 6), which realises a relative movement between zones 1 and 2. The extruded mesh in zones 5 and 6, represented by a rectangular mesh, includes the edge zones of the feeler gauge tape, the black lacquered plate of the additional heating mat, and the air. It is assumed that these zones along the extrusion axis have a minor influence on the target variables. The following Figure 5 shows the displacement of the mesh along the y-axis.

Figure 5a shows the initial state of the mesh at time t = 0 s. Figure 5b shows the extruded mesh at a relative fibre nozzle offset of 40 mm. Zone 1 moves continuously, whereas the extruded meshes in zones 5 and 6 deform. Equation (5) is used to simulate the nozzle feed rate $v_{D}$.

(5) $$v_{D}=\frac{\Delta s\;}{\Delta t}\left[\frac{\mathrm{mm}}{\min}\right]$$

Here, Δs represents the travelled path of the fibre nozzle within a discrete time step Δt. To ensure relative movement between zones 1, 5, and 6, sliding conditions are defined on the surfaces of the deformable zones so that no fixed nodes to neighbouring areas are generated during meshing.

The mesh resolutions initially assumed in Table 4 are optimised in Section 2.3.1 with the help of a convergence analysis. The model’s accuracy can be increased with more account points. However, this happens at the expense of computing time and memory requirements. Therefore, a compromise must always be found between accuracy and computing time or storage capacity.

#### 2.2.6. Physics with Initial and Boundary Conditions

**Figure 6:**
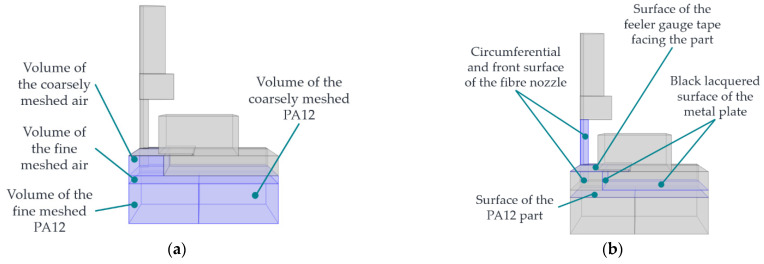
The volumes (purple) involved in the FE model in which heat conduction occurs (**a**) and the surfaces (purple) involved in the FE model which are involved in radiation exchange (**b**).

The meshing zones for the PA12 part and the air are selected in the physics module heat conduction—see Figure 6a.

The heat conduction within the heating mat is insignificant for the FE model. Therefore, only the additional heat source’s black lacquered metal plate surfaces are given a temperature boundary condition ($T_{\mathrm{HM}3}$), which can be adjusted via the model’s parameter list. This conducts heat through the air gap between the black lacquered metal plate and the PA12 part. The heat conduction from the fibre nozzle is also ensured by a temperature boundary condition ($T_{D}$). The air and the PA12 part are given an initial value ($T_{O}$) at the beginning of the simulation, corresponding to the LS machine’s preheating. Furthermore, the circumferential surfaces of the FE model receive a thermal insulation condition. On the one hand, the surfaces in the symmetry plane of the FE model must be insulated, as there are no heat flows here. On the other hand, the outer surfaces of the air region and the PA12 part are isolated. Since only a section of the black lacquered metal plate is shown in the FE model, no heat transfer from the heating mat to the surrounding process chamber occurs there.

The thermal radiation physics module in COMSOL is applied to all surfaces involved in radiation exchange—see Figure 6b. These include the surface of the black lacquered metal plate, the bottom of the feeler gauge tape, and the surfaces of the PA12 part and the fibre nozzle. The direction of the emitted radiation is determined by the opacity, which is why the radiation exchange takes place through the air areas. The total heat $Q_{\mathrm{FN}}\;$ to be transferred according to Equation (2), the multiphysics function heat transport with surface-to-surface radiation, is applied in COMSOL.

#### 2.2.7. Determination of the Depth of the HAZ

**Figure 7:**
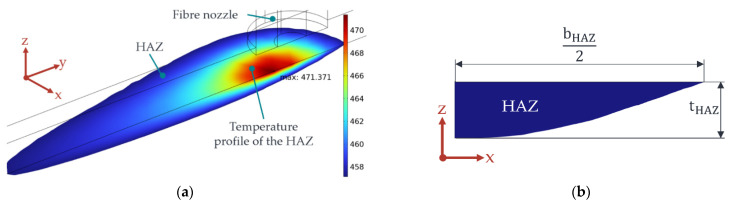
Isometric view of the evolution of the HAZ within the part when moving mesh is executed (**a**), as well as a front view of the HAZ with half width and total depth of the HAZ (**b**).

To determine the width and depth of the HAZ, the General Projection function in COMSOL is used [34]. For each discrete time step Δt of the moving mesh, the heat propagation in the PA12 part is calculated. Using the General Projection operator, starting from the centre of the HAZ, the distance along the Cartesian axes is integrated until the condition $T_{M}\geq$ 184 °C is fulfilled. A detailed description of how the General Projection Operator functions is given in [34]. The result is a value for the width and depth of the HAZ. The result of the simulation, the HAZ, is shown in Figure 7.

By default, COMSOL Multiphysics defines the time steps Δt = 3 s used to solve a time-dependent problem. The predefined settings, however, lead to irregular HAZs that do not occur in reality. The greater the feed rate of the nozzle, the worse the HAZ reproduced. To counteract this, time steps are given to the temporal solver of the FE model. A further convergence analysis examines the effect of the selected time step on the HAZ—see Section 2.3.1.

#### 2.2.8. Evaluation of Model Quality

As described in Section 2.2, a compromise must be found between model accuracy and computing time or storage capacity. The meshing zones introduced in Section 2.2.5 are increasingly refined during a convergence analysis, starting from the initially assumed mesh resolution. The resulting changes in the target value $t_{\mathrm{HAZ}}$ as a function of the number of elements/mesh resolution are presented with the help of tables and a diagram indicating the number of elements and calculation time. In addition to the standardised mesh resolutions in COMSOL (fine, finer, extra fine, and extreme fine) [33], two additional customised mesh resolutions, according to Table 5, are also used.

To determine a suitable time step ∆t of the moving mesh function, the procedure is analogous to the convergence analysis described above. The only difference is using a time step Δt instead of the mesh’s number of elements/mesh resolution. In addition to the time step of three seconds automatically defined by COMSOL, the time steps 2 s, 1 s, 0.75 s, and 0.5 s are used as time steps.

After completion of the convergence analysis, it is qualitatively checked whether the FE model correctly determines the calculated results for the depth of the HAZ. In other words, it is checked whether the physics and material parameters in the FE model correspond to the experimental results up to that point. Using a plausibility check, the results of the FE model are compared with the results of the SPD from [18]. The results of this SPD are the main effect and interaction diagrams for the investigated parameters ($T_{D},{\;h}_{\mathrm{HM}3},{\;T}_{\mathrm{HM}3},{\;h}_{D},\;\kappa ,{\;\mathrm{and}\;d}_{D,o}$). To check the plausibility of the developed FE model, the exact repetition of the SPD from [18], including the factor-level combinations, is carried out with the help of the FE model and Minitab (2022 Cloud App). A detailed description of the SPD is not given here. For this, please refer to [18]. The plausibility is checked using arrows by comparing the main effect and interaction diagrams concerning the slope. If, for example, the effect has a positive slope when changing from the first to the second level value of a factor, an ascending arrow symbol ↑ is assigned to this diagram. A descending arrow symbol ↓ is assigned in the case of a negative slope. The procedure for the other factors is analogous. Finally, the agreement of the arrow symbols, i.e., the agreement between simulation and experiment, is checked (✓). For the interactions, two arrows are used instead of one arrow.

To determine the model accuracy, the quantitative results (mean values) of the SPD are compared with the simulated results of the FE model for the same factor-level combinations. The degree of agreement, i.e., the model accuracy, ${\;e}_{\mathrm{FE}}$, between simulation and experiment is expressed in %.

### 2.3. Simulation-Based Identification of an Optimal Operating Point Range

The starting point for the simulation-based optimisation of $v_{D}$ and $t_{\mathrm{HAZ}}\;$ is the knowledge of the SPD from [18]. Section 2.1.1 describes process parameters $T_{D}$ and $v_{D,\;}\;$which must be increased to reduce the target value $t_{\mathrm{HAZ}}$ (optimisation direction). The correlation between the dependent target variable $t_{\mathrm{HAZ}}\;$ and the independent influencing variables $T_{D}$ and $v_{D}$ is required to determine an optimal operating point range.

#### 2.3.1. Determination of an Optimised Operating Point Range

To identify an optimised operating point range for $T_{D}\;$ and$\;v_{D}$, in which $t_{\mathrm{HAZ}\;}\approx$ 365 µm can be achieved, the relationship between ${\;t}_{\mathrm{HAZ}}\left(T_{D},{\;v}_{D}\right)$ is first derived with the help of a central composite design (CCD) with initially widely spaced factor levels. A CCD consists of a full factorial or partial factorial ground plan and a central star. CCD designs have an orthogonal design with a two-stage structure. The two-stage basic design can be evaluated in advance. The star can be used to create both square and cubic models. Such experimental designs are preferably used for the optimisation of target variables. Due to two independent influencing variables and a dependent target variable, the cause–effect relationship of these variables can be described in three-dimensional space. Following the Stefan–Boltzmann law as outlined in Equation (2), it is observed that alterations in temperature exhibit a nonlinear impact on the transferred heat $\;Q_{\mathrm{FN}}$. Therefore, Equation (6) serves as a fundamental framework for comprehensively depicting the linear and nonlinear influences of $T_{D}\;$ and $v_{D}$ on $\;t_{\mathrm{HAZ}\;}$.

(6) $$f\left(x,y\right)=a_{0}+a_{1}x+a_{2}y+a_{3}\mathrm{xy}+a_{4}x^{2}+a_{5}y^{2}+a_{6}x^{2}y^{2}$$

To determine the coefficients from Equation (6), assumed factor-level combinations of a central composite design (CCD) are first set and varied using the developed FE model and a linked MATLAB script, and the target value $t_{\mathrm{HAZ}}$ is determined. The initial factor levels are selected so that the results of the CCD include $t_{\mathrm{HAZ}}$ ≈ 365 µm. Information on the construction and derivation of a CCD can be found in [35]. Since the results of the FE model are not subject to scatter, the factor-level combinations are not repeated, nor are randomisation and block formation.

The following considerations determine the initial factor levels for $T_{D}\;$ and $v_{D}$ of the CCD within which the value ${\;t}_{\mathrm{HAZ}\;}$≈ 365 is expected.

- The basis for optimisation is the best operating point of the production process so far [35]. According to the results of the SPD, the best operating point range so far is based on the process understanding gained in [18], where the process parameters have a constant value of $T_{\mathrm{HM}3}=$ 190 °C and $d_{D,o}=$ 2 mm and the fibre nozzle has a planar front surface. This factor-level combination achieves an average value of $t_{\mathrm{HAZ}}$(TD = 280 °C, $h_{D}=$ 0.8 mm, and $v_{D}=$ 60 mm/min) = 52 µm. According to Equation (2), a heat quantity of approx. $Q_{\mathrm{FN}}=$ 28.7 joules is transferred to the PA12 part. This heat quantity of 28 joules, thus, forms the lower edge of the operating point range, which must be applied to generate a HAZ. The largest heat quantity of approx. $Q_{\mathrm{FN}}=$152.8 joules is reached at a factor-level combination of $t_{\mathrm{HAZ}}\;$($T_{D}=$ 310 °C, $h_{D}=$ 0.4 mm, and $v_{D}=$ 30 mm/min). A mean value $t_{\mathrm{HAZ}}\;$($T_{D}=$ 310 °C, $h_{D}=$ 0.4 mm, and $v_{D}=$ 30 mm/min) = 783 µm is achieved. Thus, the heat quantity of 152.8 joules represents the upper edge of the operating point range. The heat quantities were calculated using Equation (2), along with an averaged heat conduction coefficient for the air between the heated fibre nozzle $T_{D}\;$ and powder bed surface $T_{O}$.
- It is assumed that, for higher values of $T_{D}$ and, especially, $v_{D}$, a $t_{\mathrm{HAZ}\;}$ ≈ 365 µm can be identified as long as the amount of heat transferred to the part is within the range of 28.7 joules ≤ ${\;Q}_{\mathrm{FN}}\;$ ≤ 152.8 joules.
- As the first upper factor level (1), a value of $T_{D}$ = 360 °C and $v_{D}\;$ = 150 mm/min is initially set. The amount of heat $Q_{\mathrm{FN}}$ ($T_{D}=$ 360 °C, $v_{D}=$ 150 mm/min) thus transferred is 29.3 joules. The setting of the SPD with $T_{D}\;$ = 310 °C and $v_{D}\;$ = 60 mm/min is determined as the CCD’s lower factor level (−1). The factor-level combinations of the initial CCD are shown in Table 6.

The first four columns contain the factor-level combinations of the CCD. The last column contains the amount of heat transferred to the part. Three values are below 28 joules. Since this initial CCD intends to find a parameter constellation for $T_{D}$ and $v_{D}\;$ at which a value of $t_{\mathrm{HAZ}\;}$ ≈ 365 µm can be achieved, the CCD assumed in Table 6 is used as a first approximation. According to [18], and for reasons of process safety (Section 2.1.1), $h_{D}$ has a constant value of $h_{D}\;$= 0.6 mm for all factor-level combinations. Using the FE model and based on the factor-level combinations given in Table 6, an optimised operating point can be found for which a $t_{\mathrm{HAZ}}\left(T_{D},v_{D}\right)\approx$ 365 µm can be achieved. Based on this identified operating point, a more detailed CCD is derived, based on which the operating point determined by the FE model is to be validated experimentally.

#### 2.3.2. Experimental Validation of the Adjusted Operating Point Range

**Figure 8:**
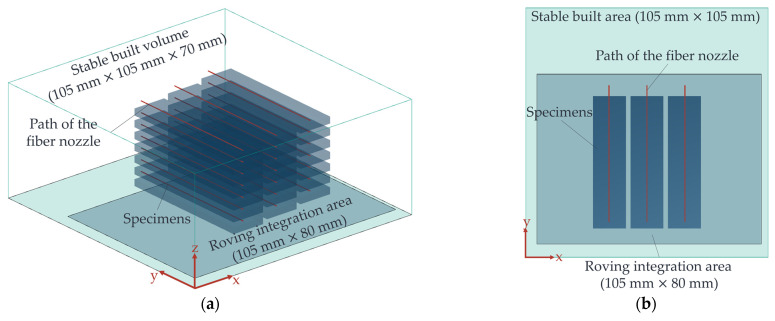
3D view of the arrangement of the specimens in the built volume of the LS machine (**a**) and a top view (**b**) with the movement path of the fibre nozzle (red lines).

The starting point for the experimental validation is the more detailed CCD determined by the FE model and adjusted concerning the factor levels. The samples with integrated HAZ are produced with the developed LS machine’s help to validate the operating point range according to the adapted CCD’s factor-level combinations. The generated HAZ is then measured in its depth. The specimens used in this study were rectangular parts with dimensions of 60 × 15 × 3 mm^3^ (l × w × h) and were constructed using PA12 material (specifically, Sintratec black). The design of these specimens was adapted from a previously established geometry described in [19]. Notably, the specimens’ thickness and width were significantly larger than the HAZ. Consequently, the heat input occurred exclusively within the generated part structure, preventing any melting of the unsintered powder. The positioning of these specimens within the powder bed of the developed LS machine is depicted in Figure 8 below.

The specimens were placed at a separation of 2 mm from each other along the x-axis. Furthermore, adjacent specimens were positioned with a 0.5 mm offset along the z-axis, corresponding to the build direction. The HAZ insertion occurred after 2.5 mm, corresponding to the 25th layer of each part. The fibre integration unit executed a rapid traverse to reach the initial position for the path of motion, indicated by the red line in Figure 8b. Subsequently; it moved at the feed rate specified in the experimental design, following which it returned to its home position via rapid traverse. Five central points were selected for each block to generate orthogonal blocks [35]. Each combination of factor levels, including two replications and the corresponding central point, was produced within a single print run, forming one block. The sequencing of experiments within two blocks was randomised using Minitab software (2022 Cloud App). The settings employed for producing the samples with the developed LS machine aligned with those described in [18]. Notably, the travel distance of the fibre nozzle, and, thus, the area subjected to melting, exceeded the dimensions of the part itself, resulting in a protrusion at the end faces of the specimens. The specimens were prepared in a manner that allowed the HAZ to be observable for evaluation to ascertain the depth of the HAZ. This was achieved by trimming off the projecting portion (a “slug”) of the melted area at the front of the specimens using a scalpel. The depth of the HAZ was then measured using a microscope (specifically, the Keyence VHM 7000), following the procedures outlined in [18].

#### 2.3.3. Influence of Roving Integration on the Depth of the HAZ

**Figure 9:**
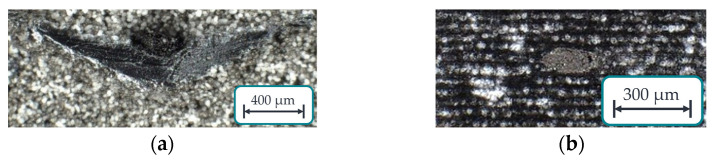
The surface of a sample with embedded 1K roving uncut (**a**) and cut open with a scalpel (**b**).

In addition to the previously outlined experimental design, an analysis was conducted to assess the impact of roving integration on the HAZ. The modified CCD was replicated while considering the influence of roving integration to accomplish this. The results were compared with those from the CCD experiments conducted without roving integration. However, given that it is challenging to visually distinguish the roving from the surrounding matrix when examining the specimen from the front (as illustrated in Figure 9a), a scalpel was employed to incise the specimen, as demonstrated in Figure 9b. This incision allowed for the measurement of the overlap of the roving [26].

**Figure 10:**
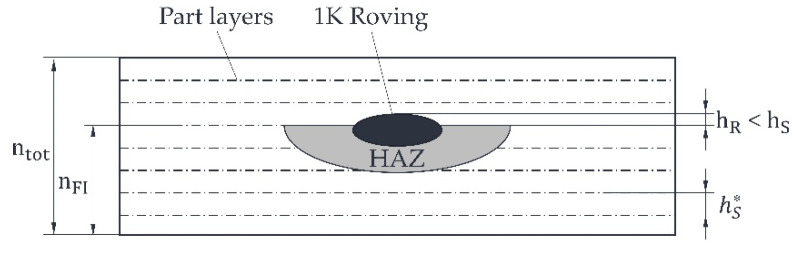
Schematic representation for calculating the roving overlap within a specimen.

In addition to the depth of the HAZ, the roving overlap is an important target value for roving integration. If the roving overlap is too high, a dragging effect of the part and the roving can occur. To determine the roving overlap, the total part thickness is first determined. Based on the known number of powder layers per part $n_{\mathrm{tot}}$ = 30, the thickness per layer${\;h}_{S}^{*}$ (≈0.095 mm) can, thus, be determined—see Figure 10. This layer thickness $h_{S}^{*}\;$ is necessary since the part will shrink during cooling. In addition, the roving overlap $h_{R}\;$can be determined from the known integration layer of the roving at $n_{\mathrm{FI}}\;$ = 25. The roving overlap is compared to the set powder layer thickness during printing and/or the movement level of the recoater at $h_{S}\;$ (= 0.1 mm) and should ideally be $h_{R}\;$ < ${\;h}_{S}$.

The machine settings employed in this study mirrored those utilised in [18]. The roving selected was a coated 1K roving (67 tex, HTA40) sourced from Teijin Limited. The chosen roving featured a coating material comprising a thermoplastic-compatible polymer dispersion known as PERICOAT AC250 [36] to facilitate adequate bonding between the fibre and matrix. The coating content was maintained at 5% [35]. Finally, the process understanding gained in this paper is validated using a demonstrator part. The demonstrator part is a battery tab gripper for handling battery electrodes within an agile battery cell production line [37].

## 3. Results and Discussion

### 3.1. Model Quality

During the convergence analysis, a change in the mesh resolution for the initially assumed coarse meshing zones 3 to 6 does not lead to a significant difference in the target value $t_{\mathrm{HAZ}}$. For meshing zones 1 and 2 and time step ∆t of the moving mesh, changes in $t_{\mathrm{HAZ}}$ could be recorded based on the convergence analysis.

**Figure 11:**
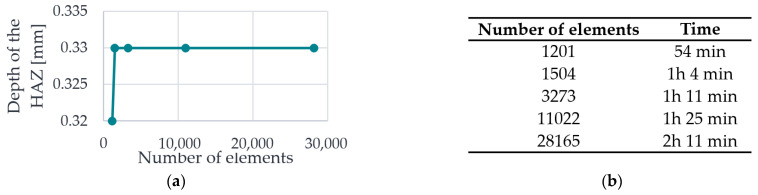
Depth of the HAZ as a function of the number of elements (**a**) and required calculation time (**b**) for meshing zone 1.

Figure 11 below shows the result of the convergence analysis for meshing zone 1.

It can be seen in Figure 11a that the target $t_{\mathrm{HAZ}}$ converges relatively early. According to Figure 11b, the required calculation time is 54 min for the smallest number of elements and 2 h 11 min for the highest number of elements. A number of elements of 110,022 are selected for this mesh (Custom 1).

**Figure 12:**
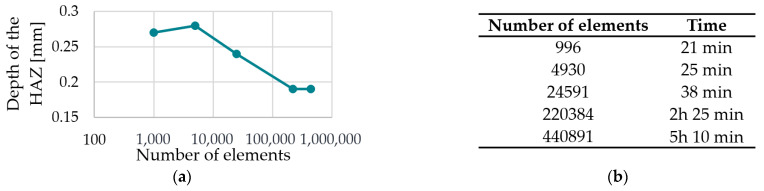
Depth of the HAZ as a function of the number of elements (**a**) and required calculation time (**b**) for meshing zone 2.

Figure 12 shows the result of the convergence analysis for meshing zone 2 (PA12).

It can be seen in Figure 12a that the target value $t_{\mathrm{HAZ}}$ appears to change extremely late, i.e., at a high number of elements. According to Figure 12b, the required calculation time is 21 min for the smallest number of elements and 5 h 10 min for the highest number of elements. Due to the significant jump between the extremely fine mesh and the Custom 1 mesh concerning $t_{\mathrm{HAZ}}\;$ of about 0.05 mm, a number of elements of 220,384 are chosen for meshing zone 2 (Custom 1).

**Figure 13:**
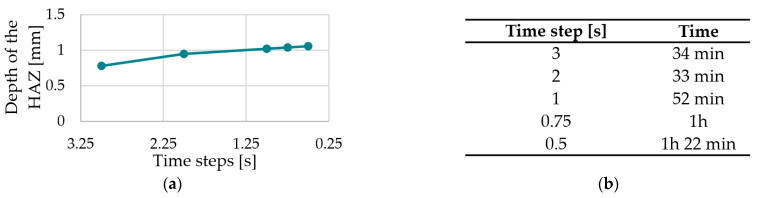
Depth of the HAZ as a function of the time step ∆t (**a**) and required calculation time (**b**) for the moving mesh.

Figure 13 shows the result of the convergence analysis for the time step ∆t.

It can be seen in Figure 13a that the target value $t_{\mathrm{HAZ}}$ does not converge even at a very high mesh resolution, i.e., at an increased number of elements. However, the changes in $t_{\mathrm{HAZ}}\;$ are still approximately 0.02 mm for the tiniest time steps. According to Figure 13b, the required calculation time is 34 min for the smallest number of elements and 1 h 22 min for the largest number of elements. Due to the relatively small change in the target value with small time steps, a time step of 0.5 s is, nevertheless, selected since it was shown during the CCD that the high values of the fibre nozzle feed rate could be better reproduced with this time step.

Table 7 summarises the mesh resolutions for the meshing zones 1–6.

In the following, the results of the plausibility check are presented. Table 8 contains the results of the plausibility check comparing the slopes of the main effects of the SPD from [18] and the slopes of the main effects of the FE model. For this purpose, the results of the FE model were evaluated using Minitab (2022 Cloud App).

It can be seen from Table 8 that the slope characteristics simulated by the FE model correspond to the slope characteristics of the main effects of the experimentally conducted SPD from [18]. Thus, it can be assumed that, at least for the main (linear) effects, the FE model provides a correct estimation for an optimised operating point range.

In Table 9, the slopes identified from the SPD for the significant interactions are compared with those from the FE model for the same interactions.

As in Table 8, it can be seen in Table 9 that the slope curves of the interactions simulated by the FE model match the slope curves of the experimentally performed SPD from [18]. Thus, in addition to the main effects, it can be assumed that the FE model provides a correct estimate for an optimised operating point range when performing the CCD in Section 3.2.

To determine the model accuracy $e_{\mathrm{FE}}$, the results of each factor-level combination of the FE model were compared with the measured values for $t_{\mathrm{HAZ}}$ from the SPD. The maximum model deviation is 22%. This results in a model accuracy of $e_{r}=$ 78%. The most probable cause for the model deviation of 22% is the unfavourable position of the factor-level combination. For this factor-level combination, the transferred heat quantity $Q_{\mathrm{FN}}$ is a value of 28.7 joules. This heat quantity corresponds to an average value of $t_{\mathrm{HAZ}}$ ($T_{D}=$ 280 °C, $h_{D}=$ 0.8 mm, $v_{D}=$ 60 mm/min, κ=Concave, $T_{\mathrm{HM}3}=$ 190 °C, and $d_{D,o}=$ 2 mm) = 52 µm. When evaluating this factor-level combination, it was found that the generated HAZ was barely visible and, thus, could not be reliably measured. This factor-level combination was repeated several times to obtain a statistically reliable result. With another factor-level combination, an average value of $t_{\mathrm{HAZ}}$ ($T_{D}=$ 310 °C, $h_{D}=$ 0.4 mm, $v_{D}=$ 30 mm/min, κ=planar, $T_{\mathrm{HM}3}=$ 190 °C, and $d_{D,o}=$ 2 mm) = 783 µm was obtained. At this setting, a heat quantity of 152.8 joules is transferred to the part. For this factor-level combination, a model accuracy of 82% could be achieved in comparison. Further causes for reduced model accuracy are listed in the following points:

- Depending on the position of the parts in the powder bed, the porosity of the parts can vary significantly due to temperature differences caused by the installed IR emitters or the additional heat source on the powder bed surface [13]. This has a direct influence on how the heat propagates within the part. The result is scattered values for the width and depth of the HAZ.
- Furthermore, the mixing ratio of the PA12 powder used has a decisive influence on the part properties. Although a mixing ratio of 60% new powder and 40% old powder was used for the SPD, according to [38], the proportion of old powder contributes to the scattering.
- Another cause of the reduced model accuracy is the possible convection flows in the air gap between the heated fibre nozzle and the part surface due to the nozzle velocity. These convection currents can influence the transferred heat quantity ${\;Q}_{\mathrm{FN}}$.
- The values in the FE model are partly based on literature values. Depending on the powder composition of the manufacturer, the material characteristics assumed in Table 3 may differ from the real material characteristics. Furthermore, the mesh resolutions have an additional influence.

In summary, it can be said that, according to the results of the plausibility check, the FE model can reflect the experiments well concerning the slope curves. A model accuracy of $e_{r}=$ 78% is classified as acceptable. Due to the proven plausibility and considering the FE model’s accuracy of $e_{r}=$ 78%, the FE model is used to identify an operating point range for $T_{D}$ and $v_{D}$, in which${\;t}_{\mathrm{HAZ}}\approx$ 365 µm occurs with high probability.

### 3.2. Operating Point Range for Roving Integration

In this section, the simulation-based derivation of an operating point range is carried out within which the rovings can be integrated into the part as reliably as possible, i.e., sufficiently deeply and with the highest possible fibre nozzle feed rate. Since the FE model does not consider the roving, an operating point range is sought with the help of the FE model by achieving a value of $t_{\mathrm{HAZ}}\;\approx$ 365 µm. $t_{\mathrm{HAZ}}\;\approx$ 365 µm is the average roving thickness. It is assumed that, at $t_{\mathrm{HAZ}}\;\approx$ 365 µm, the roving is integrated sufficiently deeply into the part without forming an interfering contour for the recoater.

#### 3.2.1. Adjustment of the Operating Point Range

**Figure 14:**
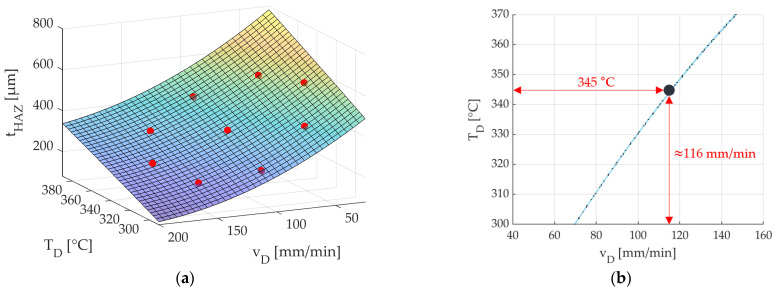
The results (red dots) of the initial CCD simulated with the FE model and the surface response diagram (**a**) derived with Minitab. Possible parameter constellations (blue line) for ${\;v}_{D}\;$and $T_{D}$ for which the condition $t_{\mathrm{HAZ}}\approx$ 365 µm applies (**b**). Selected operating point at $T_{D}=$ 345 °C (black dot).

The starting point for the simulation-based identification of an operating point range for $v_{D}\;$ and $T_{D}$ are the factor-level combinations of the CCD listed in Table 6 and initially assumed. With the help of the FE model and the MATLAB link, these factor-level combinations were set, and the regression equation and the coefficient of determination R^2^ were determined. In the following Figure 14a, the generated surface response diagram and the simulated points of the FE model can be seen in red.

It can be seen that the determined factor-level combinations from Table 6 include and can map the assumed condition ${\;t}_{\mathrm{HAZ}}\left(T_{D},v_{D}\right)\approx$ 365 µm. The regression equation calculated from Minitab (2022 Cloud App) is shown in Equation (7).

(7) $$t_{\mathrm{HAZ}}\left(T_{D},v_{D}\right)=-400+3.7v_{D}-3.4T_{D}-0.001v_{D}^{2}+0.009T_{D}^{2}-0.003v_{D}T_{D}$$

With a coefficient of determination of $R^{2}=$ 0.98, this value is close to 1. Thus, with the help of the determined regression equation, the simulated target value $t_{\mathrm{HAZ}}\left(T_{D},v_{D}\right)$ is very well reproduced. Figure 14b shows the possible parameter constellations for $T_{D}$ and$\;v_{D}$, where a value of $t_{\mathrm{HAZ}}\approx$ 365 µm is achieved. The fibre nozzle feed rate in mm/min is plotted on the x-axis, and the nozzle temperature in °C is plotted on the y-axis. The graph in Figure 14b represents the function values calculated by the regression model at constant $t_{\mathrm{HAZ}}\left(T_{D},v_{D}\right)\approx \;$365 µm.

To achieve a high fibre nozzle feed rate for $t_{\mathrm{HAZ}}\left(T_{D},v_{D}\right)\approx \;$365 µm and, thus, a reduced process time, the nozzle temperature $T_{D}\;$ must be increased at the same time according to Figure 14b so that the same amount of heat is transferred to the part. According to Figure 14b, the desired operating point range for $T_{D}$ and $v_{D}$, thus, moves to the upper right corner. Since a high fibre nozzle feed rate is sought, an initial setting at a temperature of $T_{D}=$ 345 °C is defined as the first starting point of the CCD to be adjusted, which will be used for the experimental validation. This temperature value, while maintaining the requirement $t_{\mathrm{HAZ}}\left(T_{D},v_{D}\right)\approx \;$365 µm and according to Figure 14b, corresponds to a feed rate value of approximately $v_{D}=$ 116 mm/min. This factor-level combination $T_{D}=$ 345 °C and $v_{D}=$ 116 mm/min is, thus, determined as the central point of the new, adjusted CCD. According to [35], starting from this starting point (central point), a factor-level combination is selected in the assumed optimisation direction, i.e., in the direction of a higher fibre nozzle feed rate $v_{D}$ (reduced process time for roving integration). Table 10 shows the factor-level combinations selected for the adjusted CCD with the new, more detailed operating point range.

#### 3.2.2. Experimental Validation of the FE Model in the Adjusted Operating Point Range without the Influence of Roving Integration

Based on the factor levels for the adjusted CCD in Table 10, PA12 parts were produced using the developed LS machine and the generated HAZ (without roving) was measured. The measured and FE-model-simulated values for the depth of the HAZ are listed in Table 11, together with the prevailing model deviation.

**Figure 15:**
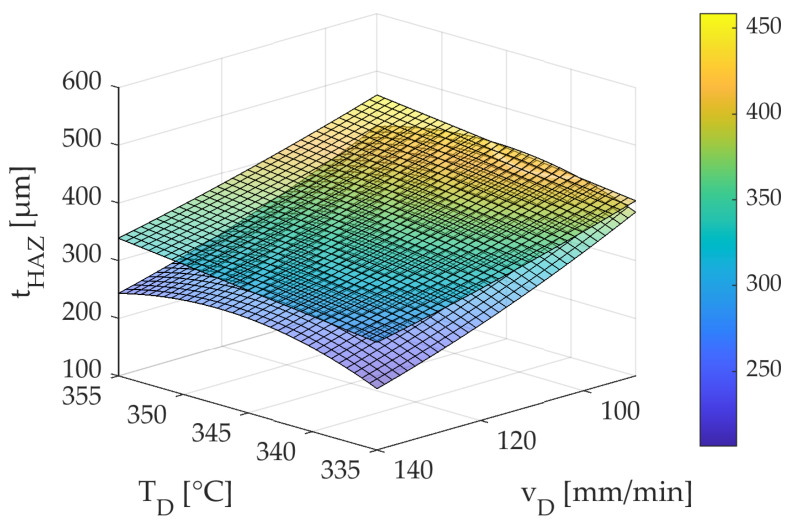
Surface response diagram for the results determined with the FE model (**top**) and the experimental results (**bottom**).

The experimental results for the depth of the HAZ are close to the simulated results. The last column shows that the relative maximum model deviation between the experiment and the FE model is max. 18%. Compared to the initial model accuracy of 22%, which was determined with the help of the SPD, the model accuracy could be reduced with the CCD. A possible reason for this could be the reduced number of factors. Compared to the SPD, where six factors are varied, with the CCD, only two factors are varied, and the rest are kept constant. This reduces the scattering influence of the factors that are held constant. Figure 15 compares the surface response diagrams of the CCD derived using Minitab for the FE model (top) and the experiments carried out (bottom).

Equation (8) shows the regression function generated by Minitab (2022 Cloud App) to describe the depth of the HAZ for the experimentally performed CCD without the influence of the roving integration (lower surface response diagram).

(8) $$t_{\mathrm{HAZ}}=46,214-274.1T_{D}-13.56v_{D}+0.3980T_{D}^{2}+0.01903v_{D}^{2}+0.0168T_{D}v_{D}$$

The standard error of the regression, S, for the depth of the HAZ was low at 32.41 µm. Therefore, the predicted deviation from the actual value was only 36.68 µm. The coefficient of determination is R^2^ = 0.78 and is rated as acceptable. Furthermore, Equation (9) shows the regression function describing the depth of the HAZ for the simulation-based CCD (upper surface response diagram).

(9) $$t_{\mathrm{HAZ}}=-400+3.7T_{D}-3.4v_{D}-0.001T_{D}^{2}+0.009v_{D}^{2}+0.003T_{D}v_{D}$$

The standard error of the regression, S, for the depth of the HAZ was also low at 32.41 µm. Therefore, the predicted deviation from the actual value was only 36.68 µm. The coefficient of determination is R^2^ = 0.98, close to 1 and rated as very good.

It can be said that the two surface response diagrams are relatively close. The surface response diagrams are further apart for high feed rate values $v_{D}$ than for lower feed values. Furthermore, it can be seen in Figure 15 that the surface response diagram for Equation (9) drops for both high and lower temperatures. The causes for deviations between the surface response diagrams could be the same as in Section 3.1. In addition, the longer sample geometry could be another reason for a reduced coefficient of determination for Equation (9). Due to the longer length of the samples compared to the samples from the SPD, the samples from the CCD may lie in areas on the powder bed where the heat distribution is no longer constant. The result is an increased pore content of the parts and a reduced value for the depth of the HAZ.

#### 3.2.3. Influence of Roving Integration on the Depth of the HAZ

The identical sets of factor levels from the modified CCD were selected to examine the impact of roving integration on the depth of the HAZ, as outlined in Table 10 [21]. Equation (10) represents the regression function derived from the CCD outcomes, used to model the HAZ depth with consideration of the roving’s influence.

(10) $$t_{\mathrm{HAZ}}=39,969-224.5T_{D}-18.14v_{D}+0.33T_{D}^{2}+0.0651v_{D}^{2}$$

**Figure 16:**
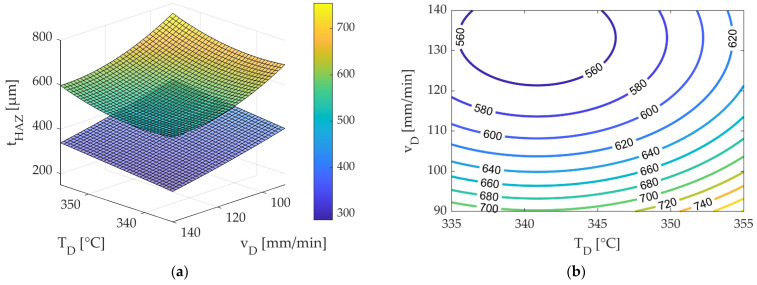
Surface response diagram of the FE model (bottom) and surface response diagram of the experimentally performed CCD with the influence of roving integration (top) (**a**). Contour diagram of the upper surface response diagram from (**a**) with contour lines (**b**).

The standard error of the regression (S) for the HAZ depth was notably low, measuring 35.69 µm. Although the coefficient of determination R^2^ of 0.72 was lower than that of the model presented in Equation (10), it is considered acceptable. In Figure 16a, a surface response plot based on Equation (9) of the FE model (excluding the influence of the roving) was compared with Equation (10) (considering the influence of the roving).

With the integration of rovings, it is evident that the depth of the HAZ becomes more substantial compared to scenarios without roving integration. Furthermore, roving integration primarily influences the depth of the HAZ, increasing it by approximately 100 µm. In comparison, the width of the HAZ experiences a marginal increment of about 32 µm, consistent with findings in [18]—this increase in depth primarily results from the heat transferred from the heated fibre nozzle to the roving. The stored heat of the roving contributes to additional melting, resulting in an average increase of 110 µm in the HAZ depth. Figure 16b presents the contour diagram for Equation (10) (CCD with roving influence). According to the contour lines in Figure 16b, the minimum depth is observed in the top-left region. In this case, it corresponds to a nozzle temperature between 335 °C and 345 °C and a feed rate between 130 mm/min and 140 mm/min. The predicted HAZ depth within this range falls below 540 µm and above 520 µm.

**Figure 17:**
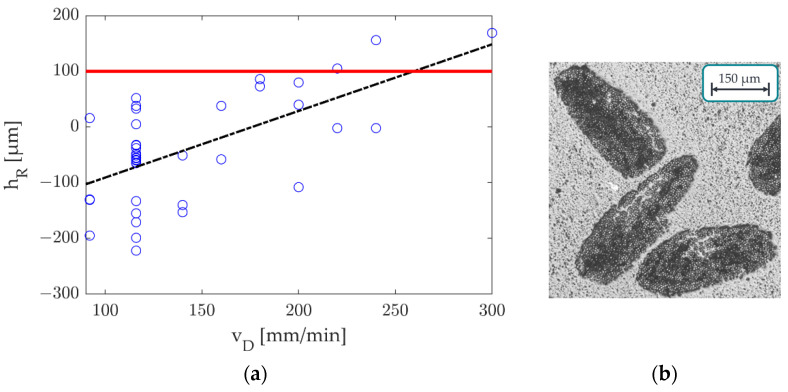
Influence of nozzle feed rate on roving overlap (**a**) and uncontrolled roving orientation within the specimen (**b**). The blue dots indicate the roving overlap and the red line in (**a**) indicates the layer thickness during printing.

To assess the reliability of the roving overlap above the integrated part level, it was observed that there was no significant influence on the nozzle feed rate within the range of 92 mm/min to 140 mm/min. Consequently, additional specimens were employed to explore the limitations of the nozzle feed rate. A nozzle temperature of 345 °C was set for these particular specimens, while the nozzle feed rate was systematically adjusted. The supplementary feed rates used were 160, 180, 200, 220, 240, and 300 mm/min, each repeated twice for reliability. Furthermore, only those derived from the modified CCD using a nozzle temperature of 345 °C were used for consistency among the specimens. Figure 17a presents the results concerning the measured roving overlaps (blue dots), the recoater’s movement level (red), and a linear trend line (black) to represent the estimated measurement points. Figure 17b illustrates the rovings’ orientation in the selected specimens’ parts.

The recoater’s movement level depicted in Figure 17a remained constant at 100 µm, aligning with the predetermined layer thickness during printing. Figure 17a provides a visual representation of the measured values’ variability. However, an increase in the nozzle feed rate resulted in the roving being less deeply embedded in the heat-affected zone (HAZ), which raised the risk of the recoater encountering the roving or potentially becoming entangled with it. Notably, while there were fewer data points for specimens with feed rates exceeding 140 mm/min, three values (representing 25% of these specimens) surpassed the 100 µm limit. Examining Figure 17b reveals significant variations in the shape and orientation of the rovings among the specimens. Some specimens exhibited flat and wide rovings, while others had tall and narrow ones. This underscores the substantial impact of orientation as a significant source of variation affecting the successful integration of rovings. The arbitrary orientation of the rovings has a significant effect on the overlap of rovings and, consequently, the reliability of the process. Process limits are established based on the conducted tests and the previously established relationships to address this issue. Increasing the nozzle feed rate while reducing the nozzle temperature led to the shallower embedding of the roving within the part. This heightened the risk of the recoater encountering and entangling the roving. Furthermore, this condition increased the likelihood of powdered material adhering to the nozzle, potentially disrupting the printing process. The powder adhering to the nozzle was observed at 335 °C with a feed rate of 160 mm/min and 345 °C with a 200 mm/min feed rate. In the experimental CCD study, a detailed examination was conducted within the temperature range of 335 °C to 355 °C for the nozzle and within the feed rate range of 92 mm/min to 140 mm/min. Notably, no disturbances were encountered during the production of 52 specimens within this range, leading to the conclusion that a process-reliable integration of fibres can be achieved within these parameters.

The following factors are potential explanations for the lower coefficients of determination and the lack of control over roving orientation:

- The entire structure of the fibre integration unit remains within the process chamber of the developed LS machine during the printing process, leading to thermal expansion and potential changes in manually set values (e.g., nozzle distance) compared to the system’s cold state. Additionally, thermal expansion of the feed spindles that position the fibre integration unit can introduce errors and alter nozzle feed rate values.
- The PLC temperature setting accuracy is approximately ±1 °C, which can contribute to variations in the measurement results.
- The inner diameter of the fibre nozzle exceeds the thickness of the roving, resulting in an increased play of the roving within the fibre nozzle. This play can lead to uncontrolled roving placement within the part.
- Other factors, such as the placement of specimens in the powder bed, the condition of the powder’s ageing, and the condition of roving delivery, may also contribute to result deviations.

#### 3.2.4. Determination of an Optimal Operating Point

All target parameters must be considered to determine the optimal operating point for the developed LS machine. Notably, the settings for nozzle temperature and nozzle feed rate exert opposing effects on the target variables. A higher nozzle feed rate proves advantageous for the width and depth of the HAZ (heat-affected zone) and processing speed. Conversely, lowering the nozzle temperature results in reduced HAZ dimensions. Conversely, a higher nozzle temperature and a lower nozzle feed rate enhance process reliability. The adjusted CCD analysis identified an optimal range between 335 °C and 345 °C at a nozzle feed rate of 130 mm to 140 mm per minute for the HAZ depth. Further reductions in either nozzle temperature or increases in nozzle feed rate should be avoided for process safety. Considering these factors, a nozzle feed rate increase enhances all three remaining target values. Therefore, a process limit of 140 mm per minute was selected for optimisation. At the same time, nozzle temperature variations have a relatively minor impact within the range of 335 °C to 345 °C; a limit of 340 °C was set to ensure greater process reliability. Considering process reliability, speed, and HAZ size, the optimised operating point corresponds to a nozzle temperature of 340 °C and a nozzle feed rate of 140 mm per minute. According to the FE model, this setting is expected to yield a HAZ width of 2638.72 µm and a HAZ depth of 523.36 µm. An overview of all settings for the optimal operating point is provided in Table 12.

In the future, several approaches can be considered to enhance process reliability and reduce processing time:

- Modifying the fibre nozzle’s inner diameter to match the roving’s shape or making it smaller can provide better control over the orientation of the rovings within the part.
- Implementing additional twisting of the rovings before coating could result in a rounder shape. This, combined with adjustments to the inner diameter of the fibre nozzle, may improve deposition accuracy within the part.

#### 3.2.5. Manufacturing of a Battery Tab Suction Gripper

**Figure 18:**
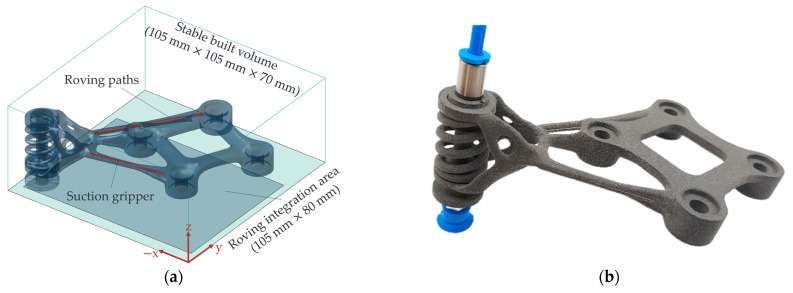
Three-dimensional view of the 3D model in the slicer app with roving paths in red (**a**) and manufactured suction gripper with integrated spring (**b**).

For the experimental validation of the identified and optimised operating points from Table 12, this section involves the production of a battery tab suction gripper with a function-integrated spring, i.e., without subsequent assembly, for agile fuel cell production. Figure 18a shows the 3D model of the suction grip with integrated roving paths (red) inside the struts to be reinforced, which was created using generative design.

In the struts, within which the rovings are to be integrated, the part has a minimum part diameter of 3.5 mm. The risk of the HAZ protruding from the part, thus affecting the struts’ overall appearance and reducing the struts’ surface quality, is, therefore, relatively high. Using the optimised operating point from Table 12, three rovings could be integrated into each strut according to Figure 18b, and, thus, the part could be successfully manufactured and reinforced. The surface of the struts differs only marginally from the rest of the part.

## 4. Conclusions

The LS machine developed for the additive manufacturing of CCFRP parts aims to combine the specific advantages of the LS process with continuous roving reinforcement. This technology enables the future production of intricate, near-net-shape functional parts with favourable matrix properties. The approach allows for load-path-oriented reinforcement with continuous rovings, offering economic benefits by eliminating the need for support structures and reducing post-processing efforts.

This study pursued the systematic optimisation of roving integration within a newly developed LS machine. This involved utilising an FE model in COMSOL and conducting experiments using a CCD. The optimisation focused on crucial aspects such as processing time, process reliability, and the HAZ’s shape. The critical findings of this study are summarised as follows:

- Using a convergence analysis and plausibility check, the developed FE model could be verified concerning model plausibility. The FE model shows the same physical behaviour as the split-plot design (SPD) in [18]. When comparing the results from the SPD and the FE model, an initial model accuracy of the FE model of 78% is achieved.
- A large percentage of the deviations between the developed FE model and the conducted experiments most likely originate in the pure LS process and the course of roving integration. Depending on the position of the parts in the powder bed, the porosity of the parts can vary significantly on the powder bed surface due to temperature differences caused by the installed IR emitters in the LS machine or the additional heat source of the fibre integration unit [20]. This has a direct influence on how the heat propagates within the part. The result is scattered values for the width and depth of the HAZ. Furthermore, the mixing ratio of the PA12 powder used has a considerable influence on the part properties. Another cause for reduced model accuracy is the occurrence of possible convection flows in the air gap between the heated fibre nozzle and the part surface due to the nozzle feed rate. These convection currents can influence the transferred heat quantity$\;Q_{\mathrm{FN}}$. The material parameters or the mesh fineness used in the FE model in COMSOL can also affect the target values and, thus, the model accuracy.
- With the help of the derived FE model and a CCD with initially widely spaced factor-level combinations, an operating point range could be identified in which $t_{\mathrm{HAZ}}\left(T_{D},v_{D}\right)\approx$ 365 µm occurs. Based on a selected operating point at $T_{D}=$ 345 °C and $v_{D}=$ 116 mm/min, a new, more detailed CCD was derived as the basis for experimental validation of the FE model.
- The adapted CCD was carried out experimentally and simulatively. In a result comparison, the model accuracy of the FE model could be reduced to 18%. The reasons for this are the reduced number of varying factors and, thus, a reduced scattering effect. The experimental regression model for the detailed CCD has a coefficient of determination of 0.78 and is close to 1. The target variable $t_{\mathrm{HAZ}}\left(T_{D},v_{D}\right)$ can thus be described relatively well by the influencing factors.
- Additional factors contributing to the lower coefficients of determination include the thermal expansion of the fibre integration unit, potential deviations in temperature settings controlled by the PLC, the interaction between the roving and the inner diameter of the fibre nozzle, and scattering effects linked to the laser-sintering process (such as material properties and specimen placement within the powder bed).
- Integrating rovings into the part results in an expanded HAZ. Specifically, the depth of the HAZ is notably increased compared to scenarios without roving integration. Roving integration primarily influences the depth, which sees an average increase of 100 µm. This heightened depth can be attributed to the heat transferred from the heated fibre nozzle to the roving. Moreover, the roving’s intrinsic heat contributes to additional melting, increasing the average HAZ depth of 110 µm.
- This study successfully demonstrated a substantial 233% increase in the nozzle feed rate, achieving a 140 mm/min rate for roving integration. Consequently, more cost-effective production of CCFRP parts in the developed LS machine becomes feasible. Furthermore, the width and depth of the HAZ were effectively reduced to 2638.72 µm −56%) and 523.36 µm (−44%), respectively. This reduction enables the integration of rovings closer to the part edges, facilitating higher fibre volume content (FVC) settings. The study, thus, provides optimised operational parameters for future research endeavours.
- However, certain limitations persist, particularly in terms of processing time. Although a 233% increase in processing time may seem substantial, the manufacturing duration for CCFRP parts with a high FVC can still be considerable. The increase in manufacturing time is only marginal for CCFRP parts, necessitating localised reinforcement in highly stressed areas with a lower FVC requirement. Additionally, it should be noted that, when rovings are placed near part edges, this may lead to protrusions of melted material or the HAZ from the part surface. Achieving a uniform surface requires the removal of these protruding materials.

Subsequent research efforts will focus on systematically augmenting the FVC and its correlated mechanical properties. In tandem with positioning rovings close to part edges, careful consideration will be given to the relative distances between rovings in both the vertical (build-up direction within the LS process) and horizontal (across the powder bed surface) axes. Tensile specimens will be fabricated to elucidate the mechanical attributes and, ultimately, reveal the potential of this LS process integrated with continuous rovings. Moreover, an investigation into the influence of nozzle temperature on roving properties is slated for exploration.

Furthermore, the established FE model can be leveraged to determine an operational point conducive to successful roving integration with alternative materials in the LS process (such as PA11, PA6, TPU, etc.). The potential applications of this LS process for CCFRP parts extend into the domain of production engineering. Notably, the cost-effective and time-efficient production of lightweight tools, purposefully tailored for industrial robot applications (e.g., gripper fingers featuring internal air channels for parallel jaw grippers or suction grippers equipped with integrated springs), stands as a promising avenue for reducing both moving masses and energy demands.

## Figures and Tables

**Table 1:** Influencing variables for keeping the part/powder bed surface temperature within the sintering window.

Symbol	Description	Unit	Setting	Value
$d_{R}$	Thickness of the 1K roving	µm	State of delivery	≈365
$h_{D}$	Distance between fibre nozzle and powder bed surface	mm	PLC	0–2
$d_{D,o}\;|{\;d}_{D,i}$	$\mathrm{Outer}\;d_{D,o}\;$and $\mathrm{inner}\;\mathrm{diameter}{\;d}_{D,i}\;$of the fibre nozzle	mm	Lathe	$d_{D,o}\geq 2$
				$d_{D,i}=0.6$
κ	The curvature of the fibre nozzle	-	Lathe	Planar/concave
$A_{\mathrm{HM}3}$	Area of the heat source (metal plate)	mm^2^	Laser cutting	≈ 22,500
$h_{\mathrm{HM}3}$	Air gap width (additional heat source to powder surface)	mm	Feeler gauge tape	0–2
$\varepsilon_{D}$	The emissivity of the fibre nozzle (black-oxidised)	-	Varnished	≈0.9
$\varepsilon_{P}$	The emissivity of the already molten part (Sintratec PA12)	-	State of delivery	≈0.9
$\varepsilon_{\mathrm{HM}3}$	The emissivity of the matte-black-painted metal plate	-	Varnished	≈0.9
$T_{D}$	Fibre nozzle temperature	°C	PLC	…400
$T_{O}$	Powder bed surface temperature	°C	PLC	…200
$T_{\mathrm{HM}3}$	Heat source temperature (metal plate)	°C	PLC	…200
$-{\dot{Q}}_{S,B}$	Heat losses due to radiation	W	Disturbance variables	
$-{\dot{Q}}_{K,B}$	Heat losses due to radiation	W		

**Table 2:** Target variables for successfully heating the part/powder bed surface within the sintering window during roving integration.

Symbol	Description	Unit
$v_{D}$	Process time expressed by the nozzle feed rate	s
$b_{\mathrm{HAZ}}$	Width of the HAZ	mm
$h_{R}$	Overlap of the roving as a measure of process reliability	mm
$t_{\mathrm{HAZ}}$	$\mathrm{Depth}\;\mathrm{of}\;\mathrm{the}\;\mathrm{HAZ}.\;\mathrm{For}\;a\;\mathrm{process}-\mathrm{safe}\;\mathrm{roving}\;\mathrm{integration},\;t_{\mathrm{HAZ}}\geq \;$350 µm is assumed.	mm

**Table 3:** Material parameters and initial values are defined in COMSOL.

Symbol	Description	Value	Unit	Source
ρ	Density of the molten part	1040	$\frac{\mathrm{kg}}{m^{3}}$	[28]
Φ	Porosity of the part	8.5	%	[29]
$T_{M}$	Melting temperature of PA12	184	°C	[30]
$T_{O}$	Temperature of the sintered part, as well as the part surface (initial value)	179	°C	[19]
$T_{A}$	The initial temperature of the air in the air gap	179	°C	[31]
$\lambda_{P}$	Thermal conductivity of the PA12 part	0.26	$\frac{W}{\left(m\cdot K\right)}$	[28]
$c_{P}$	Specific heat capacity of the PA12 part	2.66	$\frac{\mathrm{kJ}}{\left(\mathrm{kg}\cdot K\right)}$	[26]
$\varepsilon_{P}$	The emissivity of the PA12 part	0.90	-	[19]
$\varepsilon_{\mathrm{HM}3}$	The emissivity of the black lacquered metal plate of the additional heat source	0.97	-	[32]
$\varepsilon_{\mathrm{FN}}$	The emissivity of the copper fibre nozzle (oxidised)	0.76	-	[32]
$\varepsilon_{\mathrm{FGT}}$	The emissivity of the feeler gauge tape	0.85	-	[32]

**Table 4:** Individual meshing zones with initially assumed mesh resolution.

Meshing Zone	Description	Mesh Resolution
1	Common mesh for the fibre nozzle and air.	Fine
2	PA12	Fine
3	PA12	Coarse
4	PA12	Coarse
5	Extruded mesh—compressed	Coarse
6	Extruded mesh—stretched	Coarse

**Table 5:** Additional and customised meshing parameters of the convergence analysis.

Mesh Properties	Max. Element Size [mm]	Min. Element Size [mm]	Max. Element Growth Rate	Curvature Factor	Resolution of Narrow Areas
Custom 1	1	0.017	1.3	0.2	1
Custom 2	0.5	0.017	1.3	0.2	1

**Table 6:** Factor levels used for the initial CCD with abbreviations and heat quantity transferred.

$T_{D}$		$v_{D}$		$Q_{FN}$
310	(−1)	60	$\left(-1\right)$	50.9
360	(+1)	60	$\left(-1\right)$	73.2
310	$\left(-1\right)$	150	$\left(+1\right)$	20.4
360	$\left(+1\right)$	150	$\left(+1\right)$	29.3
299.7	$\left(-\surd 2\right)$	105	(0)	26.6
370.4	$\left(+\surd 2\right)$	105	(0)	44.6
335	(0)	41.4	$\left(-\sqrt{2}\right)$	89.7
335	(0)	168.6	$\left(+\sqrt{2}\right)$	22
335	(0)	105	(0)	35.3

**Table 7:** Selected mesh resolutions for meshing zones 1 to 6.

Meshing Zone	Description	Mesh Resolution
1	Common mesh for the fibre nozzle and air	Custom 1
2	PA12	Custom 1
3	PA12	Coarse
4	PA12	Coarse
5	Extruded mesh—compressed	Coarse
6	Extruded mesh—stretched	Coarse
Time step	Moving mesh	0.5 s

**Table 8:** Comparison of the slopes of the main effect diagrams between the FE model and the SPD.

Factor	Factor Level 1→Factor Level 2	Experiment Slope	Simulation Slope	Concordance
κ	(planar→concave)	↓	↓	✓
$d_{D,o}$	(2 mm→4 mm)	↑	↑	✓
$h_{D}$	(0.4 mm →0.8 mm)	↓	↓	✓
$T_{D}$	(280 °C→310 °C)	↑	↑	✓
$v_{D}$	(30 mm/min→60 mm/min)	↓	↓	✓
$T_{\mathrm{HM}3}$	(190 °C→200 °C)	↑	↑	✓

**Table 9:** Comparison of the slopes of the interaction diagrams between the FE model and the SPD.

Interaction Factor 1/Factor 2	Experiment Slope	Simulation Slope	Concordance
$\kappa \;/{\;d}_{D,o}$	↑↑	↑↑	✓
$\kappa \;/{\;h}_{D}$	↓↓	↓↓	✓
$d_{D,o}\;/{\;T}_{D}$	↑↑	↑↑	✓
$d_{D,o}\;/{\;v}_{D}$	↓↓	↓↓	✓
$v_{D}\;/{\;T}_{D}$	↓↓	↓↓	✓

**Table 10:** Factor levels used for the adjusted CCD with abbreviations.

Factor	$-\sqrt{2}$	−1	0	1	$\sqrt{2}$
$T_{D}$(°C)	335	338	345	352	355
$v_{D}$(mm/min)	92	99	116	133	140

**Table 11:** Comparison of simulated and experimental results for $t_{\mathrm{HAZ}}$ with deviation and an indication of model accuracy.

$T_{D}$		$v_{D}$		$t_{HAZ}$[µm] FE Model	${\bar{t}}_{HAZ}$[µm] Experiment	$\Delta t_{HAZ}$[µm]	Deviation [%]
338	(−1)	99	$\left(-1\right)$	388	391	4	1
352	(+1)	99	$\left(-1\right)$	426	400	−26	6
338	$\left(-1\right)$	133	$\left(+1\right)$	308	275	−33	12
352	$\left(+1\right)$	133	$\left(+1\right)$	345	292	−53	18
335	$\left(-\surd 2\right)$	116	(0)	337	295	−42	14
355	$\left(+\surd 2\right)$	116	(0)	391	336	−55	16
345	(0)	92	$\left(-\sqrt{2}\right)$	426	447	21	5
345	(0)	140	$\left(+\sqrt{2}\right)$	313	284	−29	10
345	(0)	116	(0)	365	354	−11	3

**Table 12:** Identified operating points evaluated as optimal for reproducible and process-reliable roving integration.

**Operating Points**	
$T_{D}$ (°C)	340
$v_{D}$ (mm/min)	140

## Data Availability

Not applicable.
